# Oxidative Stress in Intervertebral Disc Degeneration: New Insights from Bioinformatic Strategies

**DOI:** 10.1155/2022/2239770

**Published:** 2022-03-31

**Authors:** Yongzhao Zhao, Qian Xiang, Jialiang Lin, Shuai Jiang, Weishi Li

**Affiliations:** ^1^Department of Orthopaedics, Peking University Third Hospital, Beijing, China; ^2^Beijing Key Laboratory of Spinal Disease Research, Beijing, China; ^3^Engineering Research Center of Bone and Joint Precision Medicine, Ministry of Education, Beijing, China

## Abstract

Oxidative stress has been proved to play important roles in the development of intervertebral disc degeneration (IDD); however, the underlying mechanism remains obscure to date. The aim of this study was to elucidate the vital roles of oxidative stress-related genes in the development of IDD using strict bioinformatic algorithms. The microarray data relevant to the IDD was downloaded from Gene Expression Omnibus database for further analysis. A series of bioinformatic strategies were used to determine the oxidative stress-related and IDD-related genes (OSIDDRGs), perform the function enrichment analysis and protein-protein interaction analysis, construct the lncRNA-miRNA-mRNA regulatory network, and investigate the potential relationship of oxidative stress to immunity abnormality and autophagy in IDD. We observed a significantly different status of oxidative stress between normal intervertebral disc tissues and IDD tissues. A total of 72 OSIDDRGs were screened out for the further function enrichment analysis, and 10 hub OSIDDRGs were selected to construct the lncRNA-miRNA-mRNA regulatory network. There was a very close association of oxidative stress with immunity abnormality and autophagy in IDD. Taken together, our findings can provide new insights into the mechanism research of oxidative stress in the development of IDD and offer new potential targets for the treatment strategies.

## 1. Background

It is estimated that approximately 84% adults will suffer from the low back pain (LBP) during their lifetimes [1, 2]. Intervertebral disc (IVD) degeneration (IDD) is the principal contributor to the LBP, but the precise pathogenesis remains obscure [3]. Several mechanisms have been proved to be involved in the initiation or progression of IDD, such as mechanical stress, immunity abnormality, metabolic disorders, and oxidative stress [3–8]. Especially, oxidative stress has gotten increased attention in the pathogenesis of IDD recently [9–11].

Oxidative stress refers to an imbalance between oxidants and antioxidants in the cells and tissues, which will result in the accumulation of reactive oxygen species [6]. Recently, increasing evidence shows that oxidative stress may play vital roles in IDD by inducing the premature senescence and promoting a catabolic phenotype in human nucleus pulposus (NP) cells [9–14]. Hu et al. found that exosomes derived from bone mesenchymal stem cells could relive the compression-induced apoptosis of NP cells through inhibiting the oxidative stress [10]. Seol et al. reported that the reduction of oxidative stress by amobarbital was an effective way to prevent the IDD progression [13]. Despite a growing number of relevant studies, the underlying mechanism of oxidative stress in IDD has not been fully elucidated up to now.

Long noncoding RNA (lncRNA) refers to the noncoding RNA whose length exceeds 200 nucleotides, and it can interact with the DNA, RNA, and protein [15]. LncRNA is involved in many diseases via functioning as a competing endogenous RNA (ceRNA) to suppress the microRNA (miRNA) functions and upregulate the targeted gene expression [16–18]. However, limited research has been performed to elucidate the ceRNA network of lncRNA in the regulation of oxidative stress in IDD.

Immunity abnormality is a very crucial contributor to the development of IDD [3, 19]. The proinflammatory cytokines secreted by the NP cells and the infiltrating immune cells (IICs) are involved in the progression of IDD [5]. Autophagy is an important intracellular degradation process to maintain the homeostasis of intracellular environment by removal of damaged organelles and nonfunction proteins and cyclic utilization of degraded components [20]. The activation of autophagy may be an important protection mechanism to relieve the damage of oxidative stress and thereby prevent the progression of IDD [21, 22]. However, the underlying association of oxidative stress with immunity abnormality and autophagy in the development of IDD has not been fully understood.

In this study, we used a series of mature and recognized bioinformatic strategies to determine the following items: (1) The oxidative stress-related genes (OSRGs) involved in the pathogenesis of IDD, their main biological functions, and the potential ceRNA regulatory network; and (2) the possible relationship of oxidative stress to immunity abnormality and autophagy in IDD.

## 2. Materials and Methods

### 2.1. Data Collection and Processing

**Figure 1:**
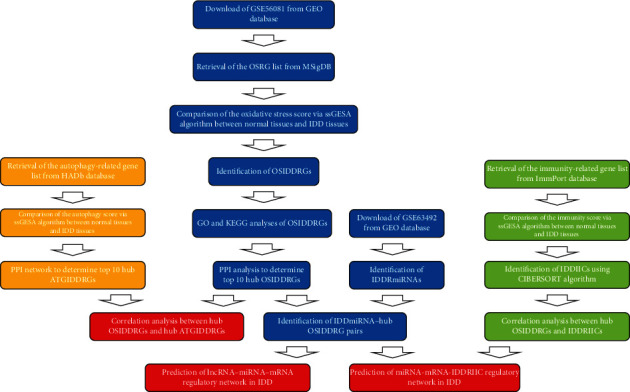
The flow chart of the bioinformatic algorithms.

This research has been approved by the institutional research ethic committee of Peking University Third Hospital, and the informed consent was exempted because all data was obtained from the common database. The flow chart of this research was shown in Figure 1. Gene expression data of mRNA and miRNA was downloaded from Gene Expression Omnibus (GEO) database (https://www.ncbi.nlm.nih.gov/geo/). The dataset used for this research was selected according to following criterions: (a) species: Homo sapiens; (b) sequencing methods: microarray data or next-generation sequencing data; and (c) samples: the nucleus pulposus in normal IVD tissues and IDD tissues. The following dataset was excluded from this research: expression data from other tissues (e.g., blood sample) and IVD tissues from subjects with scoliosis. Finally, the mRNA expression dataset GSE56081 and miRNA expression dataset GSE63492 were selected and downloaded for further analysis. The expression files of these two datasets were from 5 IDD tissues and 5 normal IVD tissues by the microarray, and the details of these tissue samples were listed in the Supplementary Table 1. The OSRG list was obtained from Gene Set GOBP_RESPONSE_TO_OXIDATIVE_STRESS in Molecular Signatures database (http://www.gsea-msigdb.org/gsea/msigdb/index.jsp) [23] (Supplementary Table 2). The immunity-related gene list was downloaded from the immunology database and Analysis Portal (ImmPort) database (https://www.immport.org/home) [24] (Supplementary Table 3). The autophagy-related gene list was downloaded from the Human Autophagy (HADb) database (http://www.autophagy.lu/index.html) (Supplementary Table 4) [25].

### 2.2. Determination of Oxidative Stress in IDD

To investigate the potential role of oxidative stress in the pathogenesis of IDD, the oxidative stress score for each tissue sample was calculated using the single sample gene set enrichment analysis (ssGSEA), which was a computational approach to explore whether a priori defined set of genes has statistical significance and concordant differences in two biological conditions for a single sample [26–28].

### 2.3. Identification of Oxidative Stress-Related and IDD-Related Genes (OSIDDRGs) and IDD-Related miRNAs (IDDRmiRNAs)

IDD-related genes were obtained using the R package limma package, with the criterion of adjusted *p* < 0.05 and fold change > 2. The OSIDDRGs were obtained with the intersection of the IDD-related genes and OSRGs using the Venn diagram. IDDRmiRNAs were obtained using the R package limma package, with the standard of *p* < 0.05 and fold change > 1.20. The volcano plot and heat map were plotted using the R package ggplot2.

### 2.4. Functional Enrichment Analysis of OSIDDRGs

Gene Ontology (GO) analysis of OSIDDRGs was conducted to interpret their biological process, cell component, and molecular function. Kyoto Encyclopedia of Genes and Genomes (KEGG) analysis was performed to explore the related signaling pathways of OSIDDRGs. The GO and KEGG analyses were both conducted using the DAVID database (https://david.ncifcrf.gov/) [29], and specific items of GO and KEGG analyses with *p* <0.05 were selected for further visualization using the R package ggplot2.

### 2.5. Protein-Protein Interaction (PPI) Analysis of OSIDDRGs

The STRING database (https://cn.string-db.org/) was a widely used tool to explore the association networks of functional proteins [30]. PPI analysis was conducted using the STRING database by imputing the OSIDDRGs into the multiple protein section, and protein pairs with score > 0.40 were selected to construct the PPI network by Cytoscape software (https://cytoscape.org/). The PPI score was calculated using the MCC method by the cytoHubba plug-in, and top 10 OSIDDRGs ranked by the PPI score were selected for further analysis.

### 2.6. Construction of ceRNA Regulatory Network of lncRNA-miRNA-mRNA

For each OSIDDRG, the correlation analysis between IDDRmiRNAs and hub OSIDDRG was performed using the Pearson test, and significantly related IDDRmiRNAs with *r* < −0.7 and *p* < 0.05 were selected for further intersection with predicted miRNAs. The predicted miRNAs for 10 hub OSIDDRGs were obtained using TargetScanHuman database (http://www.targetscan.org/vert_80/) [31]. The intersection of significantly related IDDRmiRNAs and predicted miRNAs was performed using the Venn diagram to obtain the candidate miRNAs for the construction of miRNA-mRNA pairs. Then, the upstream targeted lncRNAs for candidate miRNAs were predicted to construct the lncRNA-miRNA pairs using the Predicted model in the LncBase Predicted database (http://carolina.imis.athenainnovation.gr/diana_tools/web/index.php?r=lncbasev2/index-predicted) [32]. Ultimately, the ceRNA regulatory network of lncRNA-miRNA-mRNA was constructed and presented by the Cytoscape software.

### 2.7. Correlation Analysis between Oxidative Stress and Immunity Abnormality

The immunity score for each tissue sample was calculated using the ssGSEA algorithm based on the immunity-related gene list [5] and compared between normal IVD tissues and IDD tissues. The immune infiltration analysis was performed using the CIBERSORT database (http://cibersort.stanford.edu/) [33]. The difference of IICs was compared between normal IVD tissues and IDD tissues, and IICs with *p* < 0.05 were considered as IDD-related IICs (IDDRIICs). The correlation analysis between oxidative stress score and immunity score and the correlation analysis between 10 hub OSIDDRGs and 8 types of IDDRIICs were conducted to explore the potential relationship between oxidative stress and immunity abnormality in IDD using the Pearson test. To further elucidate the interaction between oxidative stress and immunity abnormality in the development of IDD, the miRNA-mRNA-IDDRIIC regulatory network was built by integrating the IDDRmiRNA-OSIDDRG pairs with *r* < −0.70 and *p* < 0.05 with OSIDDRG-IDDRIIC pairs with ∣*r* | >0.70 and *p* < 0.05. Ultimately, the miRNA-mRNA-IDDRIIC regulatory network was constructed and presented using the Sankey diagram.

### 2.8. Correlation Analysis between Oxidative Stress and Autophagy

The autophagy score for each tissue sample was calculated using the ssGSEA algorithm based on the autophagy-related gene list [26] and compared between normal IVD tissues and IDD tissues. To obtain the autophagy-related and IDD-related genes (ATGIDDRGs), the intersection between autophagy-related genes and IDD-related genes was conducted using the Venn diagram. Then, the PPI analysis of ATGIDDRGs was conducted to obtain the top 10 hub ATGIDDRGs using the MCC method by Cytoscape software. Furthermore, the correlation analysis between hub OSIDDRGs and hub ATGIDDRGs was conducted.

### 2.9. Statistical Analysis

Any analysis in this study was conducted by the R software 4.1.2. The ssGSEA score and percentage of IICs between normal IVD tissues and IDD tissues were compared using the Student's *t* test and *p* < 0.05 indicating there was a statistical difference between two groups. Correlation analysis in this study was performed using the Pearson test, and the correlated pair with ∣*r* | >0.70 and *p* < 0.05 was considered as statistically significant.

## 3. Results

### 3.1. Confirmation of Differential Oxidative Stress Score between Normal IVD Tissues and IDD Tissues

**Figure 2:**
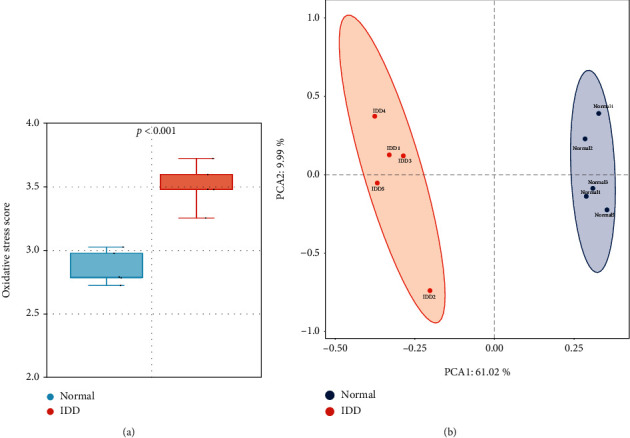
The landscape of oxidative stress in IDD. (a) the comparison of oxidative stress score between normal IVD tissues and IDD tissues; (b) PCA cluster visualizing the OSRGs.

The oxidative stress score was calculated by ssGSEA algorithm based on the expression of OSRGs, and there was a significant difference of oxidative stress score between normal IVD tissues and IDD tissues (*p* < 0.01) (Figure 2(a)). PCA analysis showed the expression of OSRGs could clearly distinguish the normal IVD tissues and IDD tissues (Figure 2(b)). Therefore, oxidative stress might play vital roles in the pathophysiology of IDD.

### 3.2. Determination of IDD-Related Genes and OSIDDRGs

**Figure 3:**
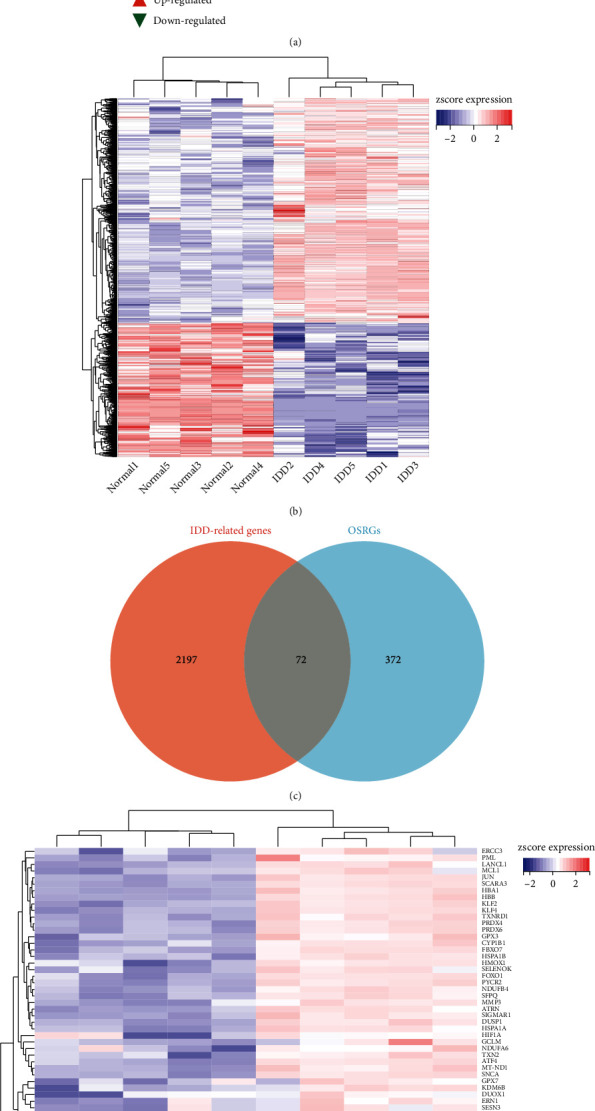
Determination of IDD-related genes and OSIDDRGs. (a) volcano plot of IDD-related genes; (b) heat map of IDD-related genes; (c) identification of 72 OSIDDRGs; and (d) heat map of 72 OSIDDRGs.

As shown in the volcano plot (Figure 3(a)), a total of 2,269 IDD-related genes were identified with the criterion of fold change > 2 and adjusted *p* < 0.05. The heat map showed the cluster analysis of these IDD-related genes could clearly distinguish the normal IVD tissues and IDD tissues (Figure 3(b)). A total of 72 OSIDDRGs were obtained by the intersection between OSRGs and IDD-related genes (Figure 3(c)), and 47 of them were upregulated and 25 were downregulated (Figure 3(d)) (Supplementary Table 5).

### 3.3. Function Analysis and PPI Analysis of OSIDDRGs

**Figure 4:**
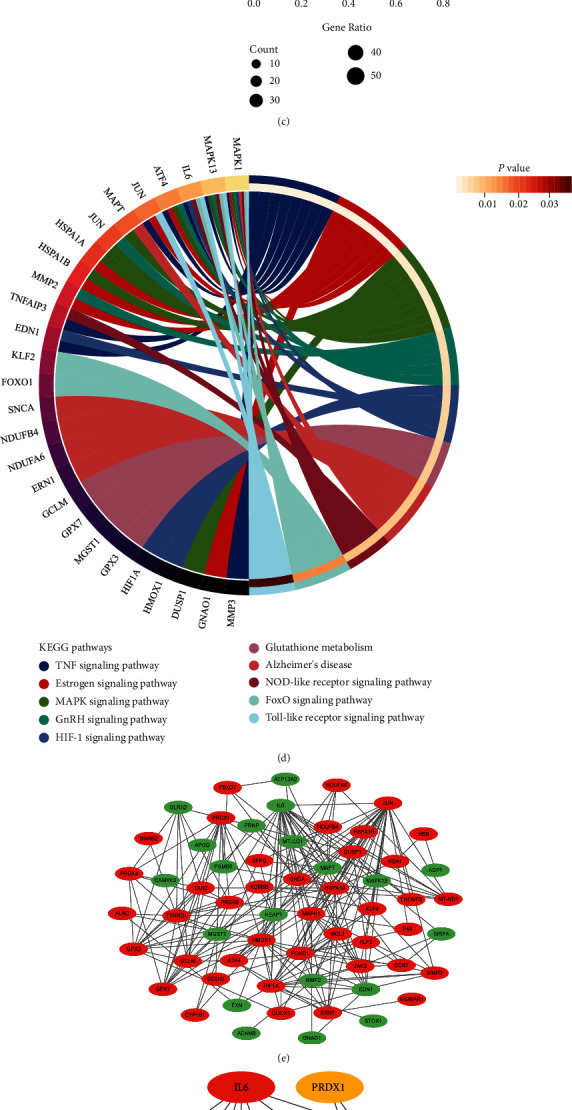
Function analysis and PPI analysis of OSIDDRGs. (a) GO_ biological process; (b) GO_cell component; (c) GO_ molecular function; (d) KEGG analysis; (e) PPI analysis; (f) top 10 hub OSIDDRGs; (g) correlation analysis among 10 hub OSIDDRGs; (h) correlation analysis between JUN and FOXO1; and (i) correlation analysis between IL6 and MAPK1.

The GO analysis, which consisted of biological process, cell component, and molecular function, was conducted to explore the biological functions of OSIDDRGs. The biological process of these OSIDDRGs was mainly enriched at the response to oxidative stress, oxidation-reduction process, positive regulation of transcription of DNA-templated, apoptotic process, response to reactive oxygen species, aging, response to hypoxia, angiogenesis, and cellular response to interleukin-1 (Figure 4(a)). These OSIDDRGs elicited the biological functions mainly at the mitochondrion (Figure 4(b)), and the most common molecular function of these OSIDDRGs was the protein binding (Figure 4(c)). KEGG analysis showed these OSIDDRGs were mainly involved in the following biological pathways: TNF signaling pathway, estrogen signaling pathway, MAPK signaling pathway, GnRH signaling pathway, HIF-1 signaling pathway, NOD-like receptor signaling pathway, FoxO signaling pathway, and Toll-like receptor signaling pathway (Figure 4(d)).

PPI analysis of 72 OSIDDRGs was conducted with a network of 77 nodes and 279 edges (PPI enrichment *p* < 0.01) (Figure 4(e)). The top 10 hub OSIDDRGs were selected, including IL6, PRDX1, MCL1, HMOX1, TXNRD1, MAPK1, HIF1A, FOXO1, JUN, and JAK2 (Figure 4(f)). The correlation analysis among top 10 hub OSIDDRGs was conducted (Figure 4(g)), and JUN-FOXO1 pair was the most positively correlated pair and IL6-MAPK1 pair (*r* = 0.98, *p* < 0.01) (Figure 4(h)) was the most negatively correlated pair (*r* = −0.86, *p* < 0.01) (Figure 4(i)).

### 3.4. Construction of lncRNA-miRNA-mRNA Regulatory Network

**Figure 5:**
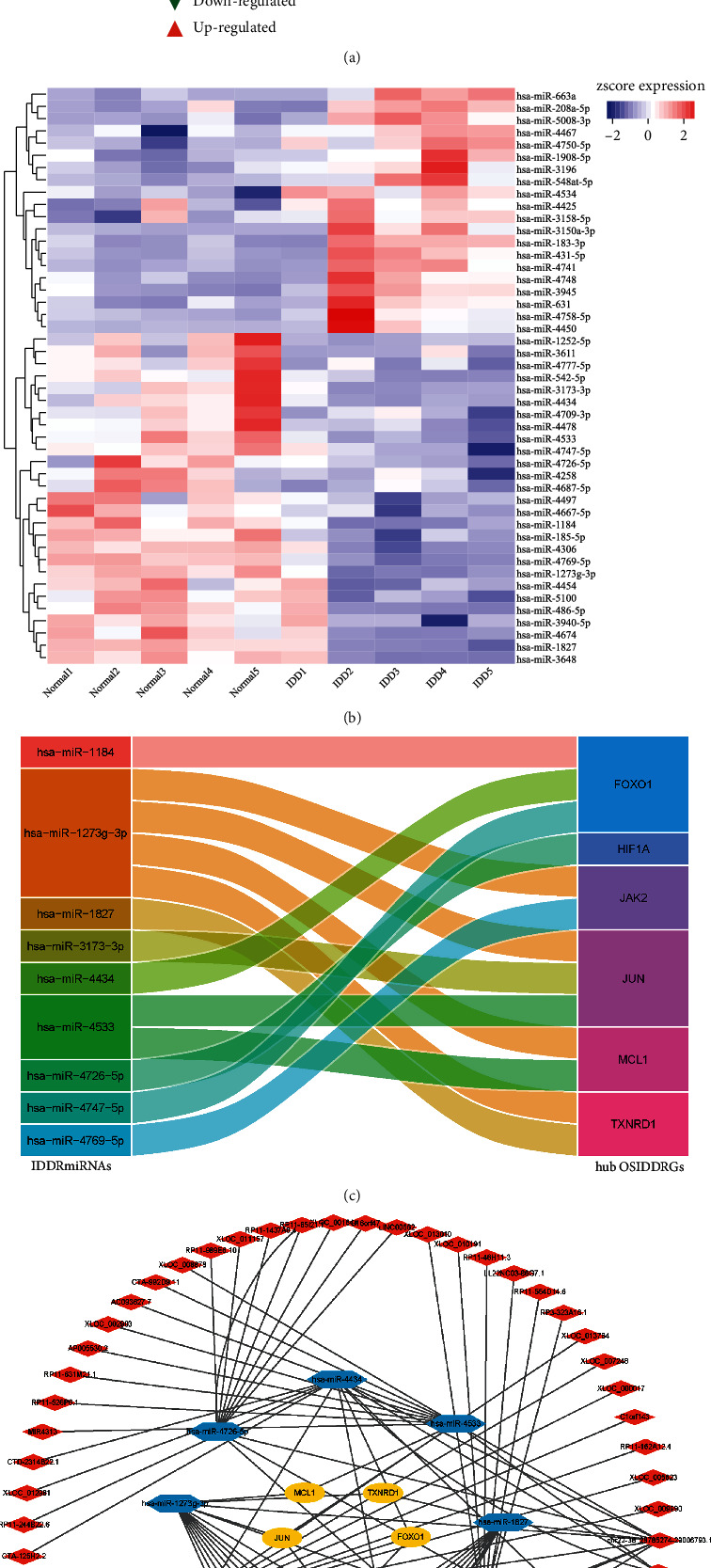
Construction of lncRNA-miRNA-mRNA regulatory network. (a) volcano plot of IDDRmiRNAs; (b) heat map of IDDRmiRNAs; (c) significant miRNA-mRNA pairs consisted of 9 IDDRmiRNAs and 6 hub OSIDDRGs; and (d) the ceRNA regulatory network consisted of 63 lncRNAs, 9 candidate miRNAs, and 6 hub OSIDDRGs.

A total of 47 IDDRmiRNAs were obtained with the standard of fold change > 1.20 and *p* < 0.05 (Figure 5(a)), and the heat map showed there was a different expression level of IDDRmiRNAs between the normal IVD tissues and IDD tissues (Figure 5(b)). For each hub OSIDDRG, the correlation analysis between the OSIDDRGs and IDDRmiRNAs was constructed. The significantly related IDDRmiRNAs (*r* < −0.70 and p < 0.05) were further used to take the intersection with the predicted miRNAs using TargetScan Human database to obtain the candidate IDDRmiRNAs (Supplementary Figure 1). Ultimately, 13 miRNA-mRNA pairs consisted of 9 IDDRmiRNAs and 6 hub OSIDDRGs were obtained (Figure 5(c)). Then, 63 targeted lncRNAs for above-mentioned 9 IDDRmiRNAs were predicted using the LncBase database, and 90 lncRNA-miRNA pairs were obtained (Supplementary Table 6). Ultimately, the ceRNA regulatory network consisting of 63 lncRNAs, 9 IDDRmiRNAs, and 6 hub OSIDDRGs was constructed (Figure 5(d)).

### 3.5. Correlation Analysis between Oxidative Stress and Immunity Abnormality in IDD

**Figure 6:**
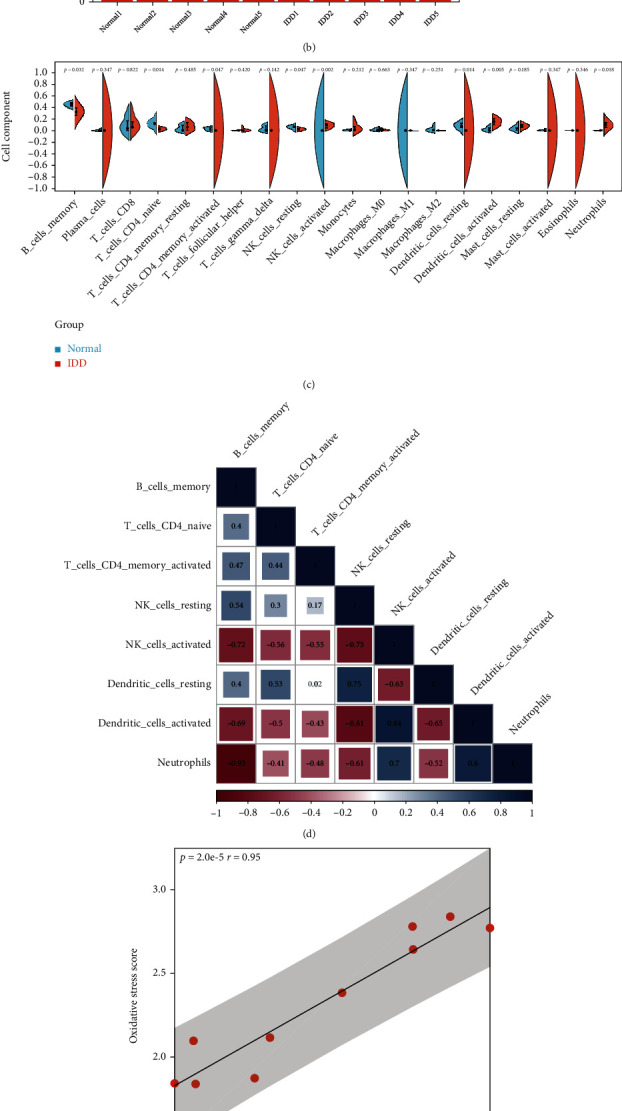
Correlation analysis between oxidative stress and immunity abnormality in IDD. (a) comparison of immunity score between normal IVD tissues and IDD tissues; (b) distribution of IICs in normal IVD tissues and IDD tissues; (c) comparison of IICs between normal IVD tissues and IDD tissues; (d) correlation analysis among 8 types of IDDRIICs; (e) correlation analysis between oxidative stress score and immunity score; (f) correlation analysis between top 10 hub OSIDDRGs and 8 types of IDDRIICs; and (g) the miRNA-mRNA-IDDRIIC regulatory work.

There was a significant difference of immunity score between normal IVD tissues and IDD tissues, which indicated that immunity abnormality played important roles in the pathogenesis of IDD (Figure 6(a)). The percentage of IICs for each sample was shown in Figure 6(b), and 8 types of IICs significantly differed between the two groups, namely, IDDRIICs (Figure 6(c)). The correlation analysis among 8 types of IDDRIICs was performed, and 7 significantly correlated pairs (*r* > 0.70, *p* < 0.05) were observed. Neutrophils-B_cells_memory was the most negatively correlated pair (*r* = −0.93, *p* < 0.01), and dendritic_cells_activated-NK_cells_activated was the most positively correlated pair (*r* = 0.84, *p* < 0.01) (Figure 6(d)). Moreover, there was a significant relationship between oxidative stress score and immunity score (*r* = 0.95, *p* < 0.01), indicating there might be a potential link between oxidative stress and immunity abnormity in the development of IDD (Figure 6(e)). To further explore the association of oxidative stress with immunity abnormity in IDD, the correlation analysis between hub OSIDDRGs and IDDRIICs was conducted, 37 significantly correlated pairs (∣*r* | >0.70, *p* < 0.05) were observed, and the neutrophils-MAPK1 pair was the most positively correlated pair (*r* = 0.89, *p* < 0.01) and B_cells_memory-JAK2 was the most negatively correlated pair (*r* = −0.84, *p* < 0.01) (Figure 6(f)). Combining the 13 miRNA-mRNA pairs with the 37 OSIDDRG-IDDRIIC pairs, a miRNA-mRNA-IDDRIIC regulatory work, which contained 9 IDDRmiRNAs, 6 hub OSIDDRGs, and 8 IDDRIICs, was constructed (Figure 6(g)).

### 3.6. Correlation Analysis between Oxidative Stress and Autophagy in IDD

**Figure 7:**
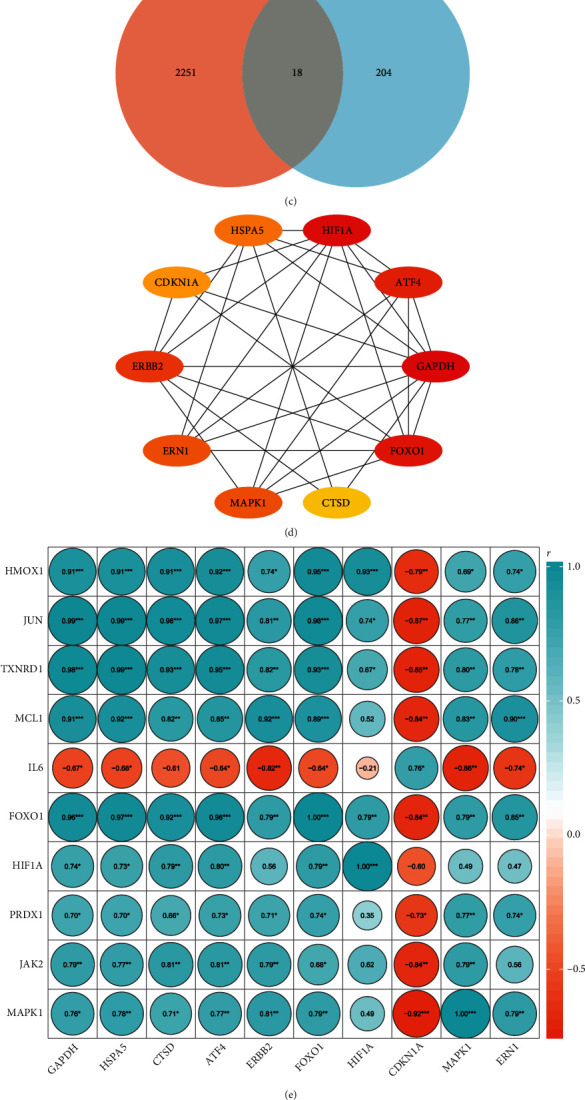
Correlation analysis between oxidative stress and autophagy in IDD. (a) comparison of autophagy score between normal IVD tissues and IDD tissues; (b) correlation analysis between oxidative stress score and autophagy score; (c) identification of 18 ATGIDDRGs; (d) top 10 hub ATGIDDRGs; (e) correlation analysis between top 10 hub OSIDDRGs and top 10 hub ATGIDDRGs; (f) correlation analysis between TXNRD1 and HSPA5; and (g) correlation analysis between MAPK1 and CDKN1A.

An obvious difference of autophagy score was observed between the two groups (*p* = 0.049) (Figure 7(a)), which manifested autophagy was involved in the development of IDD. There was a significant relationship between oxidative stress score and autophagy score (*r* = 0.80, *p* < 0.01) (Figure 7(b)), which indicated there might be a potential link between oxidative stress and autophagy in the pathogenesis of IDD. Eighteen ATGIDDRGs were obtained with the intersection between IDD-related genes and autophagy-related genes (Figure 7(c)), and top 10 hub ATGIDDRGs were selected using PPI analysis (Figure 7(d)). Then, the correlation analysis between hub OSIDDRGs and hub ATGIDDRGs was performed and 74 OSIDDRG-ATGIDDRG pairs were observed (Figure 7(e)). The TXNRD1-HSPA5 pair was the most positively correlated pair (*r* = 0.99, *p* < 0.01) (Figure 7(f)), and MAPK1- CDKN1A pair was the most negatively correlated pair (*r* = −0.92, *p* < 0.01) (Figure 7(g)).

## 4. Discussion

IDD has become a leading cause for LBP, which results in huge economic burden for the patients and medical care system [1–3]. Despite abundant researches in recent years, the definite etiology for IDD remains obscure to date [3, 5, 22]. Recently, accumulating evidence showed that oxidative stress might play important roles in the pathogenesis of IDD, but the underlying mechanism was still unclear [12, 13, 34]. In this study, for the first time, we determined the different status of oxidative stress between normal IVD tissues and IDD tissues using the ssGESA algorithm, which indicated oxidative stress had a very vital role in the IDD. Furthermore, we screened out 72 OSIDDRGs, identified their relevant biological functions, and constructed the ceRNA regulatory network using a series of bioinformatic strategies. Moreover, we also preliminarily elucidated the association of oxidative stress with immunity abnormity and autophagy in IDD. Our findings provided new insights into the researches about the oxidative stress in the development of IDD.

Through several strict bioinformatic algorithms, 10 hub OSIDDRGs were identified, including IL6, PRDX1, MCL1, HMOX1, TXNRD1, MAPK1, HIF1A, FOXO1, JUN, and JAK2. The IL6 was a famous inflammatory cytokine and could promote the initiation and progression of IDD [35]. The HMOX1 could alleviate the senescence of NP cells through inducing the autophagy [36]. The MAPK1 has been reported to be upregulated by EZH2 and promote the IDD via suppressing the miR-129-5p [37]. The HIF1A could alleviate the compression-induced apoptosis of NP-derived stem cells trough upregulating the autophagy via the HIF1A-BNIP3-ATG7 axis [38]. The FOXO1 was a vital regulator of IVD homeostasis through the direct regulation of autophagy, adaptation to hypoxia, and resistance to oxidative stress [39]. The c-Jun was found to improve the expression of TGF-*β* and promote the NP cell proliferation via reducing the apoptosis and inflammatory response [40]. The JAK2/STAT signaling pathway has been testified to protect the IVD from oxidative stress induced degeneration [41]. To our knowledge, the biological roles of PRDX1, MCL1, and TXNRD1 in IDD have not been investigated up to now. The PRDX1 has been verified to be involved in the regulation of NK cells and protective autophagy in hepatocellular carcinoma [42]. The MCL1 was a famous antiapoptotic protein and could stimulate the progression and drug resistance of thymic carcinoma [43]. The TXNRD1 was a vital ferroptosis-related gene and has been approved to promote the invasion, progression, and metastasis of hepatocellular carcinoma [44]. Based on previous studies [42–44], we made the assumption that PRDX1, MCL1, and TXNRD1 might participate in IDD progression via the regulation of oxidative stress, cell apoptosis, and immunity status, but future studies should be conducted to interpret the precise underlying mechanism. Moreover, to further explore the biological functions of OSIDDRGs, we performed the function enrichment analysis and identified several crucial KEGG pathways which might be involved in IDD. In fact, several of these identified signaling pathways have been verified in IDD, including TNF signaling pathway [45], estrogen signaling pathway [7], MAPK signaling pathway [46], HIF-1 signaling pathway [47], Toll-like receptor signaling pathway [48], and glutathione metabolism [49]. However, how the OSIDDRGs participate in IDD via these signaling pathways has not been fully understood and needs the further investigation.

LncRNA can affect the gene expression by competitively binding miRNAs, namely, ceRNA mechanism, which has been proved to play important and complicated roles in the initiation or progression of human diseases, including IDD [15, 50, 51]. Lan et al. reviewed the latest researches about the interplay between miRNAs and autophagy in the IDD and determined the vital role of miRNA-autophagy pathways in the development of IDD [52]. Similarly, in Jiang et al. study, the potential molecular mechanisms of miRNAs, lncRNAs, and circular RNAs in the progression of IDD were described based on the latest literatures, which suggested that noncoding RNAs could serve as potential targets for the IDD treatment [53]. Moreover, a bioinformatic analysis conducted by Wang et al. showed that miRNAs could function as novel targets for preventing and treating IDD by regulating their target genes [54]. In a similar manner, a meta-analysis conducted by Sherafatian et al. also observed the vital potential roles of abnormal miRNAs in the development of IDD [55]. In the current study, we constructed a strict ceRNA regulatory network of lncRNA-miRNA-mRNA, which was composed of 63 lncRNAs, 9 candidate miRNAs, and 6 hub OSIDDRGs, such as TMEM191C-miRNA-1184-FOXO1 axis and C1orf143-miRNA-4747-5p-TXNRD1. These ceRNA pathways shed new lights on the investigation of oxidative stress in IDD, which are worth our validating and investigating in the future.

Immunity abnormality is another crucial contributor to the development of IDD, and many inflammatory cytokines and IICs have been identified to aggravate the IDD [3, 5, 19]. In our study, we confirmed the differential immunity status between normal IVD tissues and IDD tissues using the ssGSEA algorithm. Subsequently, we identified 8 types of IDDRIICs and performed the correlation analysis among them. We discovered that neutrophils-B_cell_memory pair and dendritic_cells_activated- NK_cells_activated pair were the most negatively and positively correlated pair, respectively. These significantly correlated pairs were worth well of further investigation because few studies have focused on the interaction among IICs in IDD up to now. To further elucidate the potential mechanism of the link between oxidative stress and immunity abnormality in IDD, like the ceRNA regulatory network of lncRNAs, we constructed the miRNA-mRNA-IDDRIICs regulatory network, such as miR-1184- FOXO1-neutrophils axis and miR-4533-MCL1-B_cells_memory axis. We hypothesized these miRNA-mRNA pairs might serve vital roles by changing the percentage of IDDRIICs in the pathogenesis of IDD, which deserved the special attention in future researches.

Previous studies have indicated that oxidative stress might promote the IDD via the regulation of autophagy [22, 34, 56]. Tang et al. used the hydrogen-peroxide to induce the oxidative stress in IDD, and they observed an obviously increased expression of LC3-II protein, indicating that oxidative stress could promote the formation of autophagosome in NP cells [34]. Similarly, Xu et al. study showed that a treatment of 400 *μ*M tert-butyl-hydroperoxide for 6 h could also induce autophagy in NP cells by increasing the expression of LC3-II and Beclin-1 [57]. In our study, there was a significant correlation between oxidative stress score and autophagy score, suggesting a potential interaction between oxidative stress and autophagy in the pathomechanism of IDD. We identified 10 hub ATGIDDRGs through the PPI analysis and then attempted to interpret their relationship with OSIDDRGs using the Pearson analysis. Interestingly, most of hub OSIDDRGs had a significant association with hub ATGIDDRGs, indicating a close relationship between oxidative stress and autophagy in IDD. For instance, MAPK1-CDKNA1 pair was the most negatively correlated pair, which has not been fully investigated to date and deserved our major attention in the future.

Several limitations should be considered when interpreting our results. First, different from tumors, only few sequencing files of IDD were uploaded into public databases, which might be attributed to the rareness of control normal IVD tissues. As a result, the sample size of this study was relatively small, which might affect the stringency of our findings. Second, although the bioinformatic strategies used were strict and widely recognized, all findings in this study were obtained only based on the microarray data from GEO database, which needed further experimental verification. Third, although we tried to elucidate the association of oxidative stress with immunity abnormality or autophagy in IDD, the analysis performed in the current study was relatively thin, and in-depth scientific researches were needed. Forth, the retrospective design of this research would inevitably induce the bias, which might affect the reliability of results; therefore, prospective researches with the strict design were necessary to confirm our findings in the future.

Taken together, we found that normal IVD tissues and IDD tissues could be distinctly distinguished via the expression of OSRGs, and there was a significantly different oxidative stress score between the two groups, which indicated that oxidative stress might play important roles in the development of IDD. Then, through a series of strict bioinformatic strategies, the biological functions of OSIDDRGs were elucidated and 10 hub OSIDDRGs were identified (e.g., IL6, PRDX1, and MCL1). Moreover, the ceRNA network of lncRNA-miRNA-mRNA was constructed to further interpret the potential mechanism of oxidative stress in IDD. Furthermore, the relationship of oxidative stress to immunity abnormality and autophagy in the development of IDD was preliminarily evaluated. Our findings could provide new insights into the oxidative stress in IDD and deliver potential targets for the treatment of IDD.

## Data Availability

The original contributions presented in the study are included in the article/supplementary material, and further inquiries can be directed to the corresponding author/s.

## Abbreviations

LBP: Low back pain
IVD: Intervertebral disc
IDD: Intervertebral disc degeneration
NP: Nucleus pulposus
LncRNA: Long noncoding RNA
miRNA: microRNA
ceRNA: Competing endogenous RNA
IICs: Infiltrating immune cells
OSRGs: Oxidative stress-related genes
GEO: Gene Expression Omnibus
ImmPort: Immunology database and Analysis Portal database
HADb: Human autophagy database
ssGSEA: Single sample gene set enrichment analysis
OSIDDRGs: Oxidative stress-related and IDD-related genes
IDDRmiRNAs: IDD-related miRNAs
GO: Gene Ontology
KEGG: Kyoto Encyclopedia of Genes and Genomes analysis
PPI: Protein-protein interaction
IDDRIICs: IDD-related IICs.

## Supplementary Materials

Supplementary Materials Supplementary Table 1: detailed information of ten tissues samples. Supplementary Table 2: the list of oxidative stress-related genes. Supplementary Table 3: the list of immunity-related genes. Supplementary Table 4: the list of autophagy-related genes. Supplementary Table 5: the details of 72 OSIDDRGs. Supplementary Table 6: the predicted lncRNAs for miRNAs using LncBase database. Supplementary Figure 1: the intersection between significantly related IDDRmiRNAs and predicted miRNAs for each hub OSIDDRGs. (a) IL6; (b) PRDX1; (c) MCL1; (d) HMOX1; (e) TXNRD1; (f) MAPK1; (g) HIF1A; (h) FOXO1; (i) JUN; (j) JAK2.
