# Supplementary material for: Oxidative Stress in Intervertebral Disc Degeneration: New Insights from Bioinformatic Strategies
Source: Oxid Med Cell Longev. 2022 Mar 31;2022:2239770. doi: 10.1155/2022/2239770 (PMC8991415; doi:10.1155/2022/2239770)
Supplement: Supplementary Materials — Supplementary Table 1: detailed information of ten tissues samples. Supplementary Table 2: the list of oxidative stress-related genes. Supplementary Table 3: the list of immunity-related genes. Supplementary Table 4: the list of autophagy-related genes. Supplementary Table 5: the details of 72 OSIDDRGs. Supplementary Table 6: the predicted lncRNAs for miRNAs using LncBase database. Supplementary Figure 1: the intersection between significantly related IDDRmiRNAs and predicted miRNAs for each hub OSIDDRGs. (a) IL6; (b) PRDX1; (c) MCL1; (d) HMOX1; (e) TXNRD1; (f) MAPK1; (g) HIF1A; (h) FOXO1; (i) JUN; (j) JAK2. [file 2239770.f1.pdf]

Supplementary Table 1. Detailed information of ten sample tissues.

Sample number	Disc level	Pfirrmann Grade	Gender	Age, year
IDD1	L4/L5	5	Female	32
IDD2	L3/L4	5	Male	38
IDD3	L3/L4	4	Male	42
IDD4	L5/S1	5	Male	45
IDD5	L4/L5	4	Female	27
Normal1	L3/L4	1	Male	33
Normal2	L3/L4	1	Male	35
Normal3	L4/L5	1	Male	41
Normal4	L5/S1	1	Female	43
Normal5	L4/L5	1	Male	52

IDD, intervertebral disc degeneration; L, lumbar vertebra; S, sacral vertebra.

Supplementary Table 2 The list of oxidative stress-related genes

ABCC2
ABCD1
ABL1
ACOX2
ADA
ADAM9
ADIPOQ
ADNP2
ADPRS
AGAP3
AIF1
AIFM2
AKR1C3
AKT1
ALAD
ALDH3B1
ALOX5
ANGPTL7
ANKRD2
ANKZF1
ANXA1
APEX1
APOA4
APOD
APOE
APP
APTX
AQP1
ARG1
ARL6IP5
ARNT
ARNTL
ATF4
ATG7
ATOX1
ATP13A2
ATP2A2
ATP7A
ATRN
AXL
BAD
BAG5
BAK1
BCL2
BECN1
BMP7
BNIP3
BRF2
BTK
C19orf12
CA3
CAMKK2

CAPN2
CASP3
CAT
CBX8
CCL19
CCNA2
CCR7
CCS
CD36
CD38
CDK2
CFLAR
CHCHD2
CHD6
CHRNA4
CHUK
CLN8
COA8
COL1A1
CPEB2
CRK
CRYAB
CRYGD
CYBA
CYBB
CYCS
CYGB
CYP1B1
CYP2E1
DAPK1
DGKK
DHCR24
DHFR
DHFRP1
DHRS2
DIABLO
DNM2
DPEP1
DUOX1
DUOX2
DUSP1
ECT2
EDN1
EEF2
EGFR
EGLN1
EIF2S1
ENDOG
EPAS1
EPX
ERCC1
ERCC2
ERCC3

ERCC6
ERCC6L2
ERCC8
ERMP1
ERN1
ERO1A
ETFDH
ETS1
ETV5
EZH2
FABP1
FANCC
FANCD2
FBLN5
FBXO7
FBXW7
FER
FGF8
FKBP1B
FOS
FOSL1
FOXO1
FOXO3
FUT8
FXN
FYN
G6PD
GATA4
GCH1
GCLC
GCLM
GGT7
GJB2
GLRX2
GNAO1
GPR37
GPR37L1
GPX1
GPX2
GPX3
GPX4
GPX5
GPX6
GPX7
GPX8
GSKIP
GSR
GSS
GSTP1
GUCY1B1
H19
HAO1
HBA1

HBA2
HBB
HDAC2
HDAC6
HGF
HIF1A
HMOX1
HMOX2
HNRNPD
HNRNPM
HP
HSF1
HSPA1A
HSPA1B
HSPB1
HTRA2
HYAL1
HYAL2
IDH1
IL10
IL18RAP
IL6
IMPACT
INS
IPCEF1
JAK2
JUN
KCNA5
KCNC2
KDM6B
KEAP1
KLF2
KLF4
KRT1
LANCL1
LDHA
LIAS
LONP1
LPO
LRRK2
MACROH2A1
MAP1LC3A
MAP3K5
MAPK1
MAPK13
MAPK3
MAPK7
MAPK8
MAPK9
MAPKAP1
MAPT
MB
MBL2

MCL1
MCTP1
MEAK7
MELK
MET
MGAT3
MGST1
MICB
MIR103A1
MIR107
MIR132
MIR133A1
MIR17
MIR195
MIR19A
MIR21
MIR29B1
MIR34A
MIR675
MIR92A1
MIRLET7B
MMP14
MMP2
MMP3
MMP9
MPO
MPV17
MSRA
MSRB2
MSRB3
MT-CO1
MT-ND1
MT-ND3
MT-ND5
MT-ND6
MT3
MTF1
MTR
MYB
MYEF2
NAPRT
NCF1
NCF2
NCF4
NCOA7
NDUFA12
NDUFA6
NDUFB4
NDUFS2
NDUFS8
NEIL1
NET1
NFE2L1

NFE2L2
NME2
NME5
NME8
NOL3
NONO
NOS3
NOX1
NOX4
NOX5
NQO1
NR4A2
NR4A3
NUDT1
NUDT15
NUDT2
OGG1
OSER1
OXR1
OXSR1
P4HB
PAGE4
PARK7
PARP1
PAWR
PAX2
PCGF2
PCNA
PDCD10
PDE8A
PDGFD
PDGFRA
PDGFRB
PDK1
PDK2
PDLIM1
PENK
PINK1
PJVK
PKD2
PLA2R1
PLEKHA1
PLK3
PML
PNKP
PNPT1
PPARGC1A
PPARGC1B
PPIA
PPIF
PPP1R15B
PPP2CB
PPP5C

PRDX1
PRDX2
PRDX3
PRDX4
PRDX5
PRDX6
PRKAA1
PRKAA2
PRKCD
PRKD1
PRKN
PRKRA
PRNP
PRODH
PRR5L
PSEN1
PSIP1
PSMB5
PTGS1
PTGS2
PTK2B
PTPRK
PTPRN
PXDN
PXDNL
PXN
PYCR1
PYCR2
PYROXD1
RACK1
RAD52
RBM11
RBPMS
RELA
REST
RGS14
RHOB
RIPK1
RIPK3
RNF112
ROMO1
RPS3
S100A7
SCARA3
SCGB1A1
SDC1
SELENOK
SELENON
SELENOP
SELENOS
SESN1
SESN2
SESN3

SETX
SFPQ
SGK2
SIGMAR1
SIN3A
SIRPA
SIRT1
SIRT2
SLC1A1
SLC23A2
SLC25A24
SLC7A11
SLC8A1
SMPD3
SNCA
SOD1
SOD2
SOD3
SP1
SPHK1
SRC
SRXN1
STAR
STAU1
STK24
STK25
STK26
STOX1
STX2
STX4
SUMO4
TAT
TBC1D24
THG1L
TLDC2
TLR4
TLR6
TMEM161A
TNFAIP3
TOR1A
TP53
TP53INP1
TPM1
TPO
TRA2B
TRAF2
TRAP1
TREX1
TRPA1
TRPC6
TRPM2
TSC1
TXN

TXN2
TXNIP
TXNRD1
TXNRD2
UBE3A
UBQLN1
UCN
UCP1
UCP2
UCP3
VKORC1L1
VNN1
VRK2
WNT1
WNT16
WRN
XRCC1
ZC3H12A
ZNF277
ZNF580
ZNF622

Supplementary Table 3 The list of immunity-related genes IL32
ST3GAL6
KRT1
PTPRB
KCNE4
FCGR2B
XCL1
NTM
IFNA1
IL12A

VNN2
RCN3
BTLA
DOCK10
CRYBB1
SLC18A2
CXCR1
GPR35
S100A12
LYZ
WAS
FBXO6
CDH6
SPI1
PDIA3
FCGRT
CEACAM19
SLA
ANTXR2
EOMES
FCRL1
GBP5
NCF1B
LSAMP
GPR171
LIMD2
LRRC32
EIF2AK2
ZDHHC20
STAT1
IL18BP
HSPA1L
TREML1
PLEKHM3
DACT1
CD96
HLA-DRB6
FUT7
ACAP1
PGLYRP4
IL9
MEF2B

FPR3
LRP1
ADAM8
GLT8D2
IL20
GALM
ATP10A
PTPRM
RDH12
PSTPIP1
PLEKHO2
TFEC
VCAM1
CASS4
RELB
COTL1
LRCH2
ZKSCAN8
ACVR2B
TRABD2A
CD200
CISH
TNFSF12-TNFSF13
USHBP1
TNFSF12
SP110
PCOLCE
THPO
ABCD2
TYMP
MMRN2
PML
CD248
FIBIN
FBP1
TNFRSF14
CD1A
NRROS
ADAT1
TSPAN32
TTC37
N4BP2L1
LAMP5
AMIGO3
IFIT5
FAM92B
HSPA5
MLPH
HLA-F
NFKBIE
ADAP2
ARHGAP31
FCRL2

USP12
PCDH12
IRF8
TGM1
SUSD3
IL9R
SERPINF1
HS3ST2
FLT3LG
MPL
ZAP70
ENO3
LAMTOR2
FYN
BIRC6
HLA-DPB2
GBP2
ANXA1
SSTR3
IL11RA
DSP
SPRR2E
ARHGAP22
BCL6B
PCYOX1L
RBM38
LILRA5
LYPD5
PILRA
IL12RB2
NAPSB
SMAP2
LTB
FAM83A
NRP1
FLT3
RAD54L2
NLRC3
FYB1
DDX58
ECSCR
CCR9
LAIR1
COL6A2
NFAM1
ERAP2
JAK2
SIT1
IFNG
CD300E
NHLRC2
N4BP2
BTN3A2

PTCRA
DNAH8
C3
TRIM22
TMEM26
RHOH
CDK15
GGT5
HEPH
PLA1A
CD33
SCML4
NOD2
IFI44
LOXL3
IVL
CLECL1
PLXNC1
TNFRSF10C
TMEM170B
LINC00426
ADCY4
NFYB
CALHM6
JUP
PLXNA4
CHST2
EMILIN2
FABP3
LILRB1
FGD2
DOCK2
SLC1A7
HYDIN
PSMD1
PARP14
LINC00654
CSF3R
WFDC12
TNFSF13
CD300LB
IL24
NTNG2
NBEA
DCSTAMP
KIAA1755
RASSF3
TNFSF4
ARHGAP6
PSMC2
CARD8
HAMP
LAT

BIN2
CD69
SLCO5A1
GCSAML
CD74
PSMD3
CD244
FMOD
SLC15A3
STAC3
PLPPR4
DOCK8
WDFY4
RGS1
SIGLEC11
CD1B
PDE4B
PRLR
ACSM5
NOVA2
IGLL1
REST
UTS2
NECTIN3
GNGT2
LAG3
FERMT3
ASRGL1
PSAP
TMEM150B
PREX1
ICAM2
TGFBR1
CLEC14A
ADGRL4
SIGLEC17P
HTRA4
CLOCK
MMP2
EP300
SERPING1
POU2F2
SPATA13
FBN1
CCL7
IL22RA2
TNNT2
ICOS
HLA-B
IFIH1
GATM
LAP3
RASGRF2

GPR65
RNASE2
IFNK
VMO1
MINDY2
IFNW1
GZMH
FCGR1B
RGL4
PLPP7
CRYBG2
HIST1H2AG
LAMA4
CD3D
RFTN1
CDH5
CREBRF
DCBLD1
TIMP2
FCGR3A
IL17B
HCP5
TMEM229B
HEPHL1
PSMC3
ITGAM
PALM2-AKAP2
NMI
IGF1
S100A7
HCST
LCP2
LDLRAD4
NBEAL1
APOC1
TM6SF1
PRKCQ
CYBA
IL20RB
HERPUD1
TSHZ3
HLA-DRB1
FAM78A
TPK1
SYTL3
KRT14
ERAP1
PLXND1
ADAMTS9
DCHS1
IFNA2
UBASH3A
PRL

IL21R
TRPC4AP
IL4R
HIST1H2AE
COL18A1
KCNAB2
RAB20
CX3CL1
ITGAD
TBXAS1
SIGLEC7
GAB3
PCED1B-AS1
LILRA6
CD302
KCNJ8
HLA-DMA
CLEC12A
COL6A3
CXorf21
KLHDC10
LRP6
PTPN22
ACVR1B
IL4
CXCR2
DBH
TRIM61
CD40LG
PLD4
PATL2
GGT1
FCGR2A
FCGR2C
UBA7
LPL
IL22
SYT11
DOCK4
P4HA3
SDC2
TNFRSF1B
ACSL5
TSHR
HMGB1
GJB2
SCARF1
EIF2AK1
ZNF831
LCOR
BICC1
KLRK1
CTLA4

FCRL3
DYSF
ADGRF4
STXBP6
LINC00926
SPAG4
GIMAP5
TIMD4
RUFY4
DPP8
SDSL
SCUBE3
SAMHD1
CEP128
ARID5A
ZNF80
IL17RA
NFKB2
CARMIL2
PSMD11
RENBP
INHBC
SLC34A2
TTBK2
WIPF1
LILRB2
SLC25A45
MED13L
TNIK
CD28
SP100
PSMD10
PSMB10
OTOA
SDR9C7
BMP2K
NHSL2
CD200R1
GMPR
SIRPG
SOX5
TGFBRAP1
PSMD5
ARHGAP18
FAM171B
ACSS3
ADAMTS10
CD300LF
CCSER1
C5orf56
P2RX7
PLEK
FCGR1CP

MMP25
GLRX
HAVCR2
TIFAB
TNFSF8
ETV7
TRIR
HLA-DQB1
GPR18
CCNT1
LHFPL6
UBE2L6
PARP9
PDE1A
S1PR1
XKR8
GPC5
SBSN
P2RY11
KCNH2
GIPC3
PRAM1
RNASE7
IFNGR2
EDA2R
SAMD9L
CNR2
NFATC2
SLAMF1
BFSP2
FLT4
CNTF
PANX1
MILR1
IL4I1
CEACAM4
CD58
SMIM1
PSME2
GRAP
ADORA2A
TMEM200A
PVRIG
IFIT2
IFNGR1
ACTN1
KMO
ZMYND15
NCF4
BATF
MBNL3
PRTG
RTP5

NLRP12
PABPC5
FN1
GBP4
CRISPLD2
CCDC170
RHOJ
SBNO1
GBP1
ARHGAP45
BHLHA15
HMSD
ARHGAP25
EGFR
TXK
APOC2
ICK
SPRR2D
ITGBL1
GPR25
HIC1
MNDA
CD68
NKG7
HSPA6
GPR84
CCR4
JMY
UBR1
ARHGEF15
MSR1
LAIR2
DOK3
FCGR1A
ST3GAL5
KLHL6
CALR
CLEC4M
KLRC3
ARL6IP5
SCARF2
ERN1
AGAP2
COL15A1
RECK
AVPR1A
SFMBT2
MYEF2
CAMK1
KIR3DL1
GFI1
PTGER2
CD2

HECW2
ST8SIA4
FGR
MYO7A
VWF
VSTM4
NLRC4
PSMD6
LILRA1
GHRL
CD247
ADAM28
SYNDIG1
CD80
PKP3
TUBB6
IFNA21
MEI1
LTA
LIMS1
MEOX2
FNIP2
ETV3
HMCN1
TRAT1
IFIT3
PDCD1
GPR132
PSMD13
CCL3L1
IL18RAP
VPS37D
SERPINA1
XBP1
LGALS9
VIM
STAT5A
CD93
CDH20
PPP1R9A
PEAK1
RIF1
INMT
LST1
FCRL5
IL3RA
GNLY
TGM2
LCN10
HLA-G
IL18
IL7
PTPRJ

SIGLEC12
TTC21B
PNLIPRP3
LAD1
FMNL3
TNFAIP6
CXCR3
CLEC9A
TNFRSF10A
ZFPM2
PTPN6
PTH1R
RASSF5
CSF3
JCAD
FKBP11
TMEM106A
CTSS
ISG20
PIK3CD-AS1
LYN
PLCB4
GJD3
GPR82
NT5E
TESPA1
CELF2
COL6A1
CD160
TGFB1
CCR6
TAOK1
CREB3L1
KLRG1
FSTL1
OSMR
FAM168A
ARRB1
PPM1M
CIITA
EIF2AK3
MITF
NME8
PSMC4
TNFSF14
TMC8
ISG15
THEMIS2
FAM25A
IL13RA1
VAV1
SAMD3
VEGFB

TAP2
QPRT
ANO6
CYBB
KRT78
TAPBPL
CD1E
CD34
ACVR2A
MAP3K2
RAPGEF2
LAT2
EFTUD2
CSF2RA
MRPS21
CD8A
RAB33A
CSGALNACT2
TMEM79
ACOXL
ZNF185
OAS3
PIK3AP1
STAT4
IFNL2
OAS2
SIGLEC9
JSRP1
CAVIN1
IDO2
ZKSCAN1
FGF7
F5
OSCAR
CREB1
IL36G
ABCA6
GCSAM
ST6GAL1
TUBA4A
SCIMP
ANKRD36BP1
IFI27
CALHM5
ANXA6
MYO9A
EMILIN1
SELPLG
MCEMP1
NCCRP1
CACNA2D2
ICOSLG
LILRA4

PRG2
TMIGD2
KDR
SRGN
CXCR6
CCL16
VTCN1
PPFIA2
IL22RA1
THSD7A
LPAR4
PDGFRB
PNMA2
IDO1
COL6A6
PSMD2
ITGAX
APOBEC3D
RAET1G
PDCD1LG2
LAX1
CTTNBP2
ZBP1
KLHL23
C1orf162
IL10
RAB8B
SYNE1
KLK9
TRIM21
GMFG
MIR155HG
LUM
CHRD
C1orf127
POMK
LATS1
F2R
HSD11B1
SLC29A3
SERINC5
BCL2L14
CYSLTR1
CXorf65
ADAMTS14
ADGRA2
LIG3
UCP2
EMCN
CMPK2
ADAMTS16
MYO1F
KRT6C

IGDCC4
GMIP
EPCAM
CACNA2D4
HDC
CD72
NCR3
CEACAM21
PECAM1
ISLR
CRLF2
CCDC141
TRAF1
CCL1
PLCL1
IL2RB
MED13
ZNF671
C4A
MS4A14
HSPA1B
GUCY1A2
HCK
CTSL
PLVAP
C3AR1
HGF
ATP8B4
ROBO4
HFE
SH2D5
SMPDL3B
SLC24A4
ARSB
COLGALT2
PSMC6
PDE1B
DSG1
PRSS27
LEP
NECTIN2
OVOL1
CCRL2
KIR2DL3
IFI35
TMEM119
PREX2
HLA-DQB2
PCDH17
DLC1
DOK1
SLC10A2
SPN

BTN2A2
LPXN
VGLL3
PSMC5
ZNF215
GIT2
NLRP3
PTPN7
EPS8L1
CR1L
DHRS1
EBF1
ADAMTSL2
DAB2
PRRX1
PDGFB
C9orf139
ENPP4
GPR174
DENND6B
PAK5
FAT4
THEMIS
IGFLR1
KLRD1
NCF1C
KCND2
ECM2
FGF14
PDE6G
IL13
PAFAH1B2
TNFRSF6B
RBP5
RNF180
DOK6
MFAP3
TNFSF18
TVP23A
MRPL27
TBC1D10C
RFXAP
SPOCK2
FCAR
HSPA4
TCIRG1
LEPR
SH2D1A
THBS1
GPIHBP1
TLR5
IL5
GPSM3

RFXANK
FMNL1
ARHGAP9
DENND1C
OAS1
GPR55
EBF2
RNF222
NFYC
IL10RB
TMEM233
LHFPL2
RASGRP3
TIMP3
DPEP2
AMPH
IGSF6
SLIRP
ITGB2
LAMA2
SNAI3
CRTAM
LTC4S
PTGFR
FAM13C
CYP1B1
SLC8A1
PLIN3
FGD3
PARVG
CCR1
DSC1
PROM1
IL2
C1orf54
BATF2
CRCT1
CTSZ
GNS
SVOPL
ZCCHC24
ENPEP
BEX5
PRF1
PIK3R6
RUNX1T1
VENTX
IL1R1
FCRL4
FAM155A
RAET1E
NTRK1
ESAM

IL27
UBXN1
FHL5
LDB2
SIRPB1
CLEC4E
CHRNA6
GREM1
GZMK
SEC24D
NLRC5
ALOXE3
ANKRD36BP2
STARD13
TLR1
BANK1
PSMB8
IFNE
CLEC4A
PTPRO
ALOX5AP
CSF1
FLT1
CDC42SE2
HLA-A
PTAFR
GJA5
HEYL
KLRC2
SGCD
RGS13
S100A8
GPC6
TBCEL
HSPA12B
EPS8
CD207
HLA-DRA
TNFRSF13C
CCL25
LIPA
CHIT1
CD70
BTN3A1
C11orf21
ZNF835
RNASE6
BST2
STRN
EIF2AK4
IL21
INHBE
SLC39A2

ACKR3
CYFIP2
IL18R1
CYSLTR2
GIMAP2
FCGR3B
TGFB2
PAM16
RAD23B
ANKRD55
HLA-E
IL23R
RIN3
PDIA2
VASH1
AP3B1
KIR2DS4
ACE
TSLP
ARRB2
CHN1
ANGPTL2
RFX5
TNF
SLC7A7
CLEC6A
IL5RA
RAB39A
ASXL2
ARHGDIB
FNDC1
C6orf132
TNFRSF8
HLA-DQA1
CD300C
CLTB
SARDH
CD3G
CCL2
ABI3
IRF7
ITGA2B
ALDH3B1
CORIN
PDGFC
ROR1
MYO1G
RC3H2
GJA4
TLR10
SPRY1
CTSO
ALPK2

PLXDC1
LTBP2
GPR141
MAP7D1
PARP15
CHST13
SAMSN1
RASAL3
ADPRH
ITGB1
IFNAR2
CD300A
TNFAIP8L2
IFI44L
TYROBP
SEC24A
TGFBR2
CYTIP
FBXL7
ZNF620
IL1RL1
NCF1
LAPTM5
SCEL
OGFRL1
GPR137B
FLI1
PCDH18
HTR2A
KIR3DL3
CYP27A1
SLC12A3
KCNT2
IL19
NOTCH4
KLRC1
PCDHGA12
AQP10
MAN1C1
APOBEC3G
VAMP5
IL7R
LCK
BST1
SLURP1
GDF5
MVP
AMHR2
RIPOR2
FAM30A
LRRC25
DIXDC1
TLR4

SFN
PKHD1L1
S100A7A
MFRP
CCR7
SECISBP2L
PRKG1
PLA2G4E
SOX17
PMP22
TMEM176B
HIST1H3H
BTN3A3
FPR1
RPS6KA4
TNFRSF25
TENM3
CCL3
KCNK13
KL
ITGA9
LY9
RASSF4
GBGT1
LTBR
PDE3B
RAMP3
AKNA
HVCN1
ZBTB32
HLX
XAF1
SIGLEC10
HSP90AA1
RGS5
XCR1
TRPV2
GLIS3
SLCO2B1
KIR2DL1
ENOX1
GGTA1P
DOK5
APOE
ENG
RAPGEF6
WARS
PIK3R5
ZNF423
CLNK
SLAMF6
IL12B
ITGA5

UQCC2
FASLG
FCER1G
UHMK1
APLNR
ZNF660
TNFRSF4
ETS1
JAK3
CD40
CCR10
PODN
HLA-DOB
CORO1A
NCOA2
DKK2
PSMD8
CREBL2
ALOX5
MPP1
FZD4
MAP1LC3C
ERCC6L2
B2M
KCNK6
SGIP1
SON
ACVR1
LILRB3
PLEKHO1
IL12RB1
RTKN2
APOBEC3H
ZNF366
CTF1
TPSD1
IFNLR1
SNED1
CCDC102B
TGFB3
ICAM1
MYCT1
HERC6
BHLHE22
SPRR2G
GPR4
UNC93B1
MAGEL2
HS3ST1
IGSF21
SLAMF8
APOL6
LYL1

ASGR2
TSPAN4
AIM2
HSD17B14
PSMC1
ST3GAL2
PLA2G7
BCL2A1
PYHIN1
STK33
PATZ1
ANKRD17
CLCF1
FGD5
TMEM204
SIGLEC6
NID2
HLA-DPB1
CD53
SH2B3
HLA-C
IL17RB
ASXL3
MFNG
IFNA13
CD84
HNMT
RTP4
MX1
DDI2
ATE1
EPOR
DNAJC5B
GAPT
TNFRSF18
GRIN3A
DLL4
ADGRE5
SLC6A12
STK17B
ABCC9
MICAL2
MRPS12
FKBP7
BMPR1A
CLEC11A
CX3CR1
EPSTI1
MEDAG
BMPR2
CCR5
ARHGAP4
SOWAHD

FCRL6
EGF
ADGRE2
CCR3
NCR1
MXRA8
RASGRP4
PIM2
REL
NCF2
EHD2
SEM1
PTPRCAP
ITGA4
PLCB2
FCN1
SIGLEC14
AP1S2
NR1H3
PKD2L1
CSMD2
CACNA1C
BMPR1B
GNAI2
TMEM156
LILRB4
S1PR4
CNFN
TIGIT
PDGFA
TM4SF18
UNC13D
ADGRF5
CD274
SIGLEC5
CCR8
SLC25A53
MARCO
PHACTR1
ITGA1
ADAM6
TAP1
ARHGAP30
PRKAR2A
CLEC4D
LGMN
SULT1C4
HLA-DRB5
DOCK11
LILRP2
CR1
HIST1H2AM
ZNF683

ANKRD44
IL2RA
APOL3
IPCEF1
CMAHP
VEGFC
BNC2
SIRPB2
SP140
GATA3
NFKBID
CTSK
LILRA2
IKZF3
TLR8
SELL
MRO
LATS2
CPZ
RTRAF
USP51
CARD9
IL1RAP
TNFRSF10D
TTC16
GVINP1
MAN1A2
CD8B
NRXN3
IL17A
IL15
EPO
CCL4
EDA
LCE3D
CD5
SH2D3C
GRAP2
GPRIN3
GATA2
ADGRD1
CXCR5
SLC2A5
PARP12
PIP4K2A
TNFSF13B
PTGIR
IRF9
IL2RG
GNG11
TNS3
TMEM176A
EIF2A

PPL
KIRREL1
S100A16
COL4A2
NFYA
PSME3
C19orf38
LY96
DUSP16
RAB42
CD86
C5AR1
ADAMTS5
ANTXR1
ART4
P2RY2
GNA15
CDSN
P2RX5
PDGFRL
FILIP1L
CCL17
TNIP3
SLA2
ADGRE4P
DOK2
ULBP1
CXCR4
PSMD7
TBXA2R
ST6GALNAC3
AEBP1
CTSE
ASPN
PCED1B
IFNL1
HAVCR1
EFEMP2
HLA-DMB
ZC3H12D
C1orf116
COL6A5
CCL18
HRH2
HSPA8
RNF166
ASAH1
PEAK3
PSMB9
HLA-DOA
PSME1
LRRC17
ZNF469

CTSB
RASSF6
ZNF521
TLR9
ZNF827
DMXL2
GTF2A1
KLHL11
MMP16
ADCYAP1
RAB39B
APBB2
ENTPD1
ADGRG5
RGPD1
TTC24
POSTN
HSH2D
ZBTB10
ARHGAP15
NIPAL4
RUNX3
PIEZO2
KCNJ10
GPBAR1
ARRDC5
IFNAR1
MICB
IFI30
FAM83A-AS1
CCL22
CARD11
DCANP1
CDKL5
AOAH
JAKMIP1
SUCNR1
MRPL55
HLA-DQA2
KLRC4
CANX
TRAF3IP3
IL6
CETP
IRF1
NAIP
CD6
ACP5
TNFRSF1A
GPR78
GATA1
CAMK4
IFNB1

ZNF804A
SFTPB
SNX20
ROCK2
HK3
SAMD14
MR1
ITGB3
HIPK3
B3GAT1
PUS10
KIR3DL2
TIE1
TREM2
FPR2
SOD3
CLEC1A
TBX21
STARD8
IFFO1
CST7
CERKL
TAPBP
RRN3P2
LMTK2
CD226
LGI2
PSMD4
TNFRSF9
SELP
NOX4
DUOXA1
ITK
RGS18
TNFRSF11A
CYTH4
TNN
SLC45A3
FICD
IL15RA
SDS
BATF3
CALB2
ADTRP
EVPL
CD1C
CD79B
MCOLN2
CLEC5A
IL10RA
NR5A2
CDH11
ATP2A3

CTHRC1
LY86
BMP7
P2RY12
DDR2
KLK7
CLMP
TREM1
IFITM1
RNF125
KIF21B
TNFRSF17
PROCR
GYPC
CNTFR
P2RX4
CCL28
CXCL9
LGALS2
RGL1
IGSF10
FBLN5
CCL11
XPNPEP2
IL20RA
TNFRSF13B
PVR
EBI3
ANKRD22
CCL8
MRGPRF
DACT3
MZB1
KLRB1
FNBP1
ABI3BP
IL1RN
RUBCNL
CLIC5
LAMC2
ATP8A1
CD163L1
CXCL2
MGAT4A
SPARCL1
ALDH1A1
UBD
PBX4
SULT2B1
JAM2
GIMAP7
EDAR
SALL2

STAB1
HSP90AB1
LYPD3
MS4A7
P2RY13
PRKAR2B
LILRB5
GIMAP8
VEGFA
P2RY8
SFRP2
PPM1H
PFDN2
C1QTNF7
AKAP5
OASL
KCNMB1
KCTD12
VPREB3
CD180
FRZB
TESC
HSPA2
PDE3A
MGAT5
COL14A1
SULT1C2
ADA2
MRVI1
CTSG
RELN
ACTA2
CCL19
AQP1
FCER2
MMP14
CCL21
TMEM140
EVI2B
GSDMA
ADAM12
KIR2DL4
AQP9
CD163
RASSF2
FCMR
NXPE4
CD48
GZMA
FUCA1
CXCL8
ZEB2
STAP1

DMKN
PLPP3
FBLN2
MAN1A1
TNFRSF12A
CLIP3
PTPRC
PTGIS
CD7
RCAN2
P2RY10
CPVL
EDNRB
SSC5D
AZGP1
SELENBP1
HSPA1A
GLIPR2
TLR3
PLEK2
RAB37
MS4A2
CTSW
WNT2
CHAC1
IL23A
C15orf48
PPP1R16B
PIK3CG
CLIC2
FCRLA
STRIP2
HLA-DPA1
PADI2
NAALADL1
F13A1
FCGBP
KIT
TNFRSF11B
SHE
TPSG1
MAP4K1
KRT16
LRRC15
ARHGEF6
TRPV3
CPNE5
MS4A4A
LOXL2
TAGAP
CPXM1
TLR7
P2RX1

TSPAN11
IL1B
DPEP1
GZMM
OLFML1
FAM49A
FAS
CDH3
RHOD
C16orf54
COL11A1
SPRR1B
COL5A2
ADRM1
COL3A1
BDKRB2
PSMD14
ADAMTS12
KCNA3
ADRA2A
PRELP
KCNN4
HAPLN3
KRT6A
CD209
SPRR1A
SPARC
ENPP2
DENND2A
PLA2G2D
PARM1
PDGFRA
COL4A1
ACVRL1
SIGLEC1
ABCB1
CD36
MSRB3
CSF1R
FGL2
PLPP4
INHBB
RARRES2
APOBR
PLAC9
ICAM3
IL11
CCL20
ZEB1
SPIB
CFP
INPP5D
KIAA1549

SNTB1
CCL23
COL5A3
BGN
FOXP3
ABCA9
SLC17A9
MMP3
LRMP
FAP
PAG1
ABCA8
PGM5
ITGB7
TMEM47
BHLHE41
HPGDS
FSTL3
SIGLEC8
RSAD2
CMA1
COL12A1
TNFRSF10B
CILP
OLFML3
CD14
BLK
SERPINE1
AKAP12
FCER1A
CD79A
CLIC3
CD3E
AIF1
SPNS3
CHRDL1
AMH
HHLA2
ENPP3
C7
KITLG
CASP5
UBXN11
THY1
LCP1
MET
COLEC12
CD22
BTK
FAM107A
MAOB
DERL3
GNG2

C1QA
PLCL2
ERP27
CXCL11
RIMKLA
RTN1
SLIT2
DUSP4
S100B
IRF4
SLAMF7
RAI2
HTRA3
SECTM1
BMP2
CCR2
CXCL1
JCHAIN
BEND5
SKAP1
CXCL16
CCDC69
MRC1
CCDC80
RIN1
SPON1
ULBP2
UNC5C
TNFSF9
CLEC3B
SULF1
GPR183
TCEAL7
ADAMTS4
COL1A2
COL5A1
RNASE1
APOBEC3A
ITM2A
LYVE1
LIME1
DPT
GNG7
CXCL3
CD27
CXCL10
CCL13
TRANK1
ANK2
VSIG4
INHBA
CLEC4G
PDZRN3

IL1A
JAML
PRKCB
PKIB
NEGR1
VEGFD
TNFSF11
ITGAL
CD4
VCAN
IL16
CXCL6
A2M
OLFML2B
FSCN1
NCKAP1L
SHISAL1
HCLS1
AMPD1
DHRS9
COL10A1
KCNN3
TCL1A
AGTR1
VSIR
AFF3
ANGPTL1
PEG3
CXCL5
E2F5
DCN
PTGDS
LIFR
S100A9
MGP
CAV1
PPP1R13L
SCN7A
GIMAP6
RASGRP2
RAET1L
DDX60
ACKR1
MFAP4
LSP1
SDCBP2
PDZK1IP1
GAS7
EDNRA
EVI2A
GJB3
XCL2
MEF2C

IQGAP2
PNOC
ITGA8
AHCYL2
TIMM50
TGFBI
CALD1
CCL5
PPBP
ADAMTS2
APBB1IP
NAP1L3
DAAM2
RASL12
TNFSF15
MPEG1
C1QC
CR2
CMKLR1
CSF2
FOLR2
FERMT2
JAM3
COL1A1
SNRPF
OMD
IL1R2
TPSB2
ADAMDEC1
TOX
NUGGC
CPA3
RCSD1
CCL26
IKZF1
ARHGEF37
TEK
CD37
GIMAP1
CCL14
KRT6B
CLEC10A
COL8A1
GPR157
IL6R
CXCL12
C10orf99
GFRA3
IL6ST
NDNF
GZMB
SH2D2A
GHR

C2
IFI6
S100A2
ULBP3
GJB5
LIF
CNRIP1
IFITM3
FLVCR2
GALNT15
FAM177B
ITGA11
GPR15
OSM
SLIT3
MS4A6A
SLC11A1
PTGDR
MS4A1
CD1D
OGN
MMP12
ACHE
OLR1
MMP9
HPGD
TCN2
SASH3
CD19
GPR34
MMP1
GIMAP4
TNFSF10
C1QB
P2RY14
MMRN1
SAMD9
CCL24
AREG
CD52
LMOD1
POU2AF1
AOC3
PLEKHN1
THBS2
CXCL13
CYSRT1
CSF2RB
CPED1
TPSAB1
CCL15
MXD1

Supplementary Table 4 The list of autophagy-related genes AMBRA1
APOL1
ARNT
ARSA
ARSB
ATF4
ATF6
ATG10
ATG12
ATG16L1
ATG16L2
ATG2A
ATG2B
ATG3
ATG4A
ATG4B
ATG4C
ATG4D
ATG5
ATG7
ATG9A
ATG9B
ATIC
BAG1
BAG3
BAK1
BAX
BCL2
BCL2L1
BECN1
BID
BIRC5
BIRC6
BNIP1
BNIP3
BNIP3L
C12orf44
C17orf88
CALCOCO2
CAMKK2
CANX
CAPN1
CAPN10
CAPN2
CAPNS1
CASP1
CASP3
CASP4
CASP8
CCL2
CCR2
CD46

CDKN1A
CDKN1B
CDKN2A
CFLAR
CHMP2B
CHMP4B
CLN3
CTSB
CTSD
CTSL1
CX3CL1
CXCR4
DAPK1
DAPK2
DDIT3
DIRAS3
DLC1
DNAJB1
DNAJB9
DRAM1
EDEM1
EEF2
EEF2K
EGFR
EIF2AK2
EIF2AK3
EIF2S1
EIF4EBP1
EIF4G1
ERBB2
ERN1
ERO1L
FADD
FAM48A
FAS
FKBP1A
FKBP1B
FOS
FOXO1
FOXO3
GAA
GAA
GABARAP
GABARAP
GABARAPL1
GABARAPL1
GABARAPL2
GABARAPL2
GAPDH
GAPDH
GNAI3
GNAI3
GNB2L1

GNB2L1
GOPC
GOPC
GRID1
GRID1
GRID2
GRID2
HDAC1
HDAC6
HGS
HIF1A
HSP90AB1
HSPA5
HSPA8
HSPB8
IFNG
IKBKB
IKBKE
IL24
IRGM
ITGA3
ITGA6
ITGB1
ITGB4
ITPR1
KIAA0226
KIAA0652
KIAA0831
KIF5B
KLHL24
LAMP1
LAMP2
MAP1LC3A
MAP1LC3B
MAP1LC3C
MAP2K7
MAPK1
MAPK3
MAPK8
MAPK8IP1
MAPK9
MBTPS2
MLST8
MTMR14
MTOR
MYC
NAF1
NAMPT
NBR1
NCKAP1
NFE2L2
NFKB1
NKX2-3

NLRC4
NPC1
NRG1
NRG2
NRG3
P4HB
PARK2
PARP1
PEA15
PELP1
PEX14
PEX3
PIK3C3
PIK3R4
PINK1
PPP1R15A
PRKAB1
PRKAR1A
PRKCD
PRKCQ
PTEN
PTK6
RAB11A
RAB1A
RAB24
RAB33B
RAB5A
RAB7A
RAC1
RAF1
RB1
RB1CC1
RELA
RGS19
RHEB
RPS6KB1
RPTOR
SAR1A
SERPINA1
SESN2
SH3GLB1
SIRT1
SIRT2
SPHK1
SPNS1
SQSTM1
ST13
STK11
TBK1
TM9SF1
TMEM49
TMEM74
TNFSF10

TP53
TP53INP2
TP63
TP73
TSC1
TSC2
TUSC1
ULK1
ULK2
ULK3
USP10
UVRAG
VAMP3
VAMP7
VEGFA
WDFY3
WDR45
WDR45L
WIPI1
WIPI2
ZFYVE1

Supplementary Table 5 The details of 72 OSIDDRGs

Gene symbol	Log(fold change)	Adjusted p-value
APOD	-6.43	1.74E-07
MMP2	-2.88	1.37E-03
PRR5L	-2.15	2.72E-04
GLRX2	-1.95	1.06E-04
CAMKK2	-1.90	3.18E-06
MAPT	-1.83	5.97E-05
ADAM9	-1.83	5.51E-03
TRPM2	-1.75	2.68E-04
IL6	-1.64	2.54E-02
KEAP1	-1.60	2.49E-02
PSMB5	-1.51	4.14E-03
AQP1	-1.47	5.81E-05
GNAO1	-1.27	2.33E-03
SLC23A2	-1.27	2.42E-02
FXN	-1.23	2.74E-02
EDN1	-1.23	4.40E-02
STOX1	-1.21	5.37E-04
PRNP	-1.15	1.10E-02
MAPK13	-1.15	1.81E-02
PLEKHA1	-1.13	2.68E-02
ATP13A2	-1.11	1.46E-03
MGST1	-1.08	4.13E-04
SIRPA	-1.07	1.65E-02
VRK2	-1.03	2.40E-02
MT-CO1	-1.01	1.11E-03
PRDX1	1.00	3.25E-02
DUSP1	1.04	4.61E-05
ALAD	1.13	2.37E-02
NDUFB4	1.14	1.36E-04
DUOX1	1.15	1.17E-02
MCL1	1.20	3.02E-04
GPX7	1.21	1.44E-02
ERCC3	1.21	1.23E-03
HIF1A	1.22	2.18E-02
NDUFA6	1.23	6.24E-03
KDM6B	1.23	1.35E-03
GCLM	1.23	2.33E-03
ERN1	1.26	3.92E-03
GPX3	1.27	6.01E-05
HSPA1A	1.28	5.48E-05
SELENOK	1.30	2.31E-04
TNFAIP3	1.30	1.67E-02
MMP3	1.33	2.48E-04
PML	1.37	3.76E-05
JAK2	1.38	6.99E-03
SIGMAR1	1.39	7.67E-06
HSPA1B	1.41	1.27E-04
TXN2	1.50	4.91E-04
SESN3	1.58	4.50E-03
HMOX1	1.60	6.15E-04
SFPQ	1.63	3.76E-05

FBXO7	1.65	2.15E-05
MAPK1	1.69	6.99E-03
MSRB2	1.71	2.68E-04
ATRN	1.71	1.14E-05
CCR7	1.77	3.86E-03
SNCA	1.83	1.69E-06
PRDX4	1.87	5.23E-06
LANCL1	2.11	9.86E-06
PRDX6	2.14	2.42E-06
JUN	2.16	8.92E-07
ATF4	2.45	1.05E-05
FOXO1	2.66	2.27E-05
KLF2	2.68	1.34E-06
PYCR2	2.82	4.33E-06
MT-ND1	3.18	2.91E-07
KLF4	3.46	1.83E-07
CYP1B1	3.48	1.96E-05
SCARA3	3.52	1.90E-07
TXNRD1	3.56	6.01E-08
HBA1	6.39	3.36E-10
HBB	8.19	1.39E-09

Supplementary Table 6 The predicted lncRNAs for miRNAs using LncBase database

hsa-miR-1184	hsa-miR-1273g-3p	hsa-miR-1827
chr22-38_28785274-29006793.1	chr22-38_28785274-29006793.1	chr22-38_28785274-29006793.1
KCNQ1OT1	XLOC_007321	LOC100190986
XLOC_003397	RP11-1094M14.14	RP13-580B18.4
XLOC_010295	ZNF561-AS1	RP11-92B11.3
AC006548.28	RP4-734G22.3	LINC00960
TMEM191C	MIR4534	RP11-80F22.15
LINC00475	AC006548.28	RP11-458F8.4
TMEM191C	RP11-2E17.2	XLOC_006242
XLOC_001668	RP11-509A17.3	MIR4534
XLOC_003405	XLOC_006684	RP11-80F22.15

hsa-miR-3173-3p	hsa-miR-4434	hsa-miR-4533
chr22-38_28785274-29006793.1	chr22-38_28785274-29006793.1	chr22-38_28785274-29006793.1
CTB-176F20.3	KCNQ1OT1	MIR4313
LINC00665	CTB-176F20.3	KCNQ1OT1
MIR6818	CTA-125H2.2	RP11-526P6.1
LINC00665	AC006548.28	RP11-631M21.1
RP11-96K19.4	RP11-244B22.6	AP005530.2
AC006548.28	XLOC_012981	XLOC_002903
AC010226.4	CTD-2314B22.1	AC093627.7
FAM201A	MIR4534	CTA-992D9.11
XLOC_011696	KCNQ1OT1	XLOC_008878

hsa-miR-4726-5p	hsa-miR-4747-5p	hsa-miR-4769-5p
RP11-989E6.10	chr22-38_28785274-29006793.1	chr22-38_28785274-29006793.1
chr22-38_28785274-29006793.1	XLOC_013784	XLOC_013010
XLOC_011157	KCNQ1OT1	KCNQ1OT1
RP11-1437A8.4	AC006548.28	XLOC_010191
RP11-85I21.1	XLOC_007248	AC006548.28
XLOC_001644	XLOC_000017	RP11-46H11.3
AC006548.28	C1orf143	LL22NC03-86G7.1
RP11-85I21.1	RP11-162A12.4	MIR4534
C16orf47	XLOC_005623	RP11-554D14.6
LINC00662	XLOC_009990	RP3-323A16.1

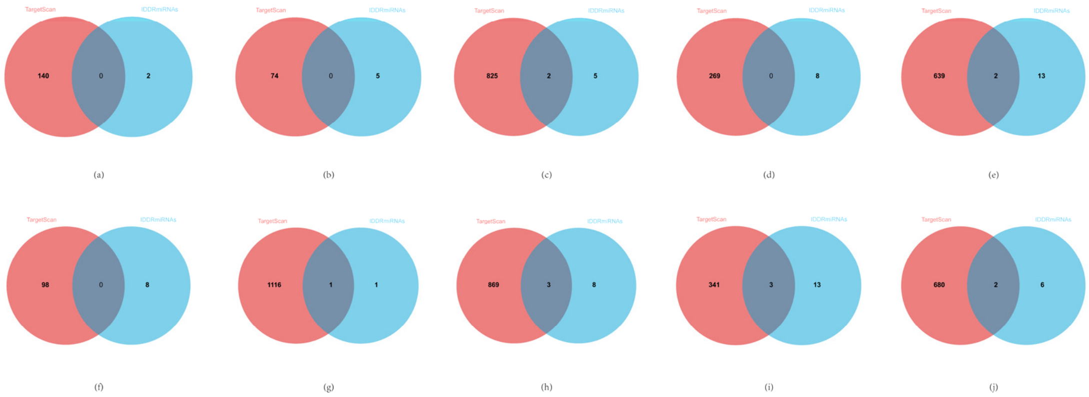

Supplementary Figure 1 The intersection between IDDRmiRNAs and predicted miRNAs using the TargetScanHuman. (a) IL6; (b) PRDX1; (c) MCL1; (d) HMOX1; (e) TXNRD1; (f) MAPK1; (g) HIF1A; (h) FOXO1; (i) JUN; (j) JAK2.
